# Connection of Nicotine to Diet-Induced Obesity and Non-Alcoholic Fatty Liver Disease: Cellular and Mechanistic Insights

**DOI:** 10.3389/fendo.2017.00023

**Published:** 2017-02-10

**Authors:** Amiya P. Sinha-Hikim, Indrani Sinha-Hikim, Theodore C. Friedman

**Affiliations:** ^1^Division of Endocrinology, Metabolism and Molecular Medicine, Department of Internal Medicine, Charles R. Drew University of Medicine and Science, Los Angeles, CA, USA; ^2^David Geffen School of Medicine at University of California, Los Angeles, CA, USA

**Keywords:** nicotine, high-fat diet, obesity, oxidative stress, non-alcoholic fatty liver disease

## Abstract

Non-alcoholic fatty liver disease (NAFLD) poses a serious health hazard affecting 20–40% of adults in the general population in the USA and over 70% of the obese and extremely obese people. In addition to obesity, nicotine is recognized as a risk factor for NAFLD, and it has been reported that nicotine can exaggerate obesity-induced hepatic steatosis. The development of NAFLD has serious clinical complications because of its potential progression from simple hepatic steatosis to non-alcoholic steatohepatitis (NASH), liver cirrhosis, and hepatocellular carcinoma. Multiple mechanisms can be involved in nicotine plus high-fat diet-induced (HFD) hepatic steatosis. Emerging evidence now suggests that nicotine exacerbates hepatic steatosis triggered by HFD, through increased oxidative stress and hepatocellular apoptosis, decreased phosphorylation (inactivation) of adenosine-5-monophosphate-activated protein kinase and, in turn, up-regulation of sterol response-element binding protein 1-c, fatty acid synthase, and activation of acetyl-coenzyme A-carboxylase, leading to increased hepatic lipogenesis. There is also growing evidence that chronic endoplasmic reticulum stress through regulation of several pathways leading to oxidative stress, inflammation, perturbed hepatic lipid homeostasis, apoptosis, and autophagy can induce hepatic steatosis and its progression to NASH. Evidence also suggests a central role of the gut microbiota in obesity and its related disorders, including NAFLD. This review explores the contribution of nicotine and obesity to the development of NAFLD and its molecular underpinning.

## Introduction

In 2009, approximately 20% (~60 million) of Americans smoked and about ~88 million non-smokers were exposed to secondhand smoke (1). Unless dramatic progress is made in diminishing the initiation and increasing cessation of combustible tobacco product use, a billion of preventable death will occur in twenty-first century worldwide (2). Thus cigarette smoking needs to be viewed as a chronic disease, and in addition to research on the difficult problem of smoking cessation, research also needs to be conducted on the detrimental effects of chronic cigarette use. The prevalence of smoking was 31.1% among persons below the federal poverty level (1), so smoking should be considered a health disparity. Cigarette smoking is the leading preventable cause of death and disability worldwide (3, 4). Smoking is a major risk factor for chronic obstructive pulmonary disease and lung cancer and devastating cardiovascular disease (CVD), such as myocardial infarction, sudden death, stroke, and peripheral vascular disease (5–8), with a dose–response correlation between CVD morbidity and mortality and the number of cigarettes smoked (8). Furthermore, usages of nicotine only formulations, such as transdermal patches, nicotine gum, and electronic cigarettes, in particular, are increasing (9, 10). The lack of targeted and effective strategies to control tobacco consumption contribute to large burden of cardiovascular disorders in low- and middle-income people worldwide, where CVD has become the leading cause of morbidity and mortality (8). Moreover, smoking leads to substantial financial costs to society. Between 2009 and 2012, smoking cost the USA approximately $289–332.5 billion, with 46–53% of this amount spent on adult medical care and the rest due to loss of workplace productivity (4). The negative effects of smoking, thus, leads to reduced quality of life and loss of life and can lead to personal and national financial burden. The health risk associated with smoking can be exaggerated by obesity (11, 12).

Nicotinic acetylcholine receptors (nAChRs) are a family of ionotropic receptor proteins formed by five homologous or identical subunits and are involved in signal transduction between neurons and muscle cells (10, 13, 14). nAChRs are divided into muscle (α1, β1, γ/ε, and δ) and neuronal nAChRs (α 2–10 and β 2–4) (10, 14, 15). Neuronal nAChRs are further subdivided into those that form homomeric receptors when expressed in heterologous systems (α7-10) and those that form heteromeric receptors (α2-6 and β2-4) in different combinations (10, 14, 15). nAChRs are also expressed in various tissues, including adipocytes, pancreatic beta cells, hepatocytes, myocytes, and cardiomyocytes (16–19). The nAChRs, which are activated by nicotine or its metabolites cotinine, can activate various signaling pathways that can alter cellular metabolic homeostasis (10). This review discusses emerging evidence of contribution of nicotine when combined with obesity to the development of hepatic steatosis and insights into the molecular mechanisms by which nicotine contributes to non-alcoholic fatty liver disease (NAFLD).

### NAFLD Is Highly Prevalent in Obese Individuals and Can Be Exaggerated by Smoking

Non-alcoholic fatty liver disease is the most common liver disorder and is associated with metabolic syndrome and diabetes mellitus. It includes the whole spectrum of fatty liver, ranging from simple steatosis to steatohepatitis [non-alcoholic steatohepatitis (NASH)], which can progress to liver cirrhosis and hepatocellular carcinoma (20–22). Data from the Framingham Heart Study showed that fatty liver is characterized by dysglycemia and dyslipidemia independent of visceral adipose tissue (23). There is increasing evidence that smoking can also contribute to NAFLD. Multiple logistic regression analysis from a retrospective follow-up study over a 10-year period, involving 2,029 Japanese subjects, demonstrated that cigarette smoking (adjusted odd ratio 1.91; 95% confidence interval 1.34–2.72) is an independent risk factor for NAFLD (24). A statistically significant association between smoking history and severity of liver fibrosis was demonstrated in a large multicenter cohort of 1,091 subjects with biopsy-proven NAFLD (25). Of further importance, the health risk associated with smoking, whether passive or active, is exaggerated by obesity, and smoking and obesity are the leading causes of morbidity and mortality worldwide (11, 12). The life expectancy of an obese smoker is 13 years less than that of a normal-weight non-smoker (11). Furthermore, smoking lowers the body weight and body mass index (BMI), which make many people reluctant to quit smoking (11).

In the United States, 72% of the adult male population is overweight or obese out of which 11% have a BMI of 35 kg/m^2^ and 4% a BMI of at least 40 kg/m^2^ (26). Obese men are at a higher risk to develop atherosclerosis, coronary heart disease, diabetes, hypertension, dyslipidemia, and NAFLD (27). NAFLD, in turn, can also be an independent risk factor of atherosclerosis and CVD (28, 29). Currently, 34% of the general population and over 75% of the obese and extremely obese individuals are estimated to have hepatic steatosis (30). Hispanics have the highest prevalence of hepatic steatosis followed by Caucasians and then African-Americans (31).

### Mechanisms Linking Nicotine to NAFLD

The hallmark of NAFLD is accumulation of triglycerides (TG) in the hepatocytes (steatosis). Multiple mechanisms have proposed to explain the accumulation of TG in the liver, including (i) increased dietary fat intake, (ii) excess free fatty acid (FFA) delivery from lipolysis of white adipose tissue, (iii) increased *de novo* lipogenesis, (iv) reduced fatty acid β-oxidation, and (v) reduced fat export in the form of very low-density lipoprotein (VLDL) (21, 32). The precise molecular mechanisms of the pathogenesis of steatosis and its progression to NASH are not well understood. AMP-activated protein kinase (AMPK) is a central regulator of lipid homeostasis and mediates suppression of lipogenic gene expression, such as acetyl-coenzyme A-carboxylase (ACC) and fatty acid synthase (FAS) through inhibition of sterol regulatory element binding protein-1c (SREBP1-c) and carbohydrate response-element binding protein (ChREBP) (33–35). ACC is the rate determining enzyme for the synthesis of malonyl-CoA, both a critical substrate for fatty acid biosynthesis and a potent inhibitor of fatty acid oxidation (33). AMPK can phosphorylate and inactivate ACC leading to inhibition of *de novo* fatty acid and cholesterol synthesis (33). AMPK can also increase the activity of malonyl-CoA decarboxylase to further decrease malonyl-CoA levels (33). Lipogenesis is further regulated by glucose, which activates ChREBP, which, in turn, activates gene expression of most enzymes involved in lipogenesis (21).

### Two-Hit or Multiple-Hit Hypothesis

Steatosis can prime the liver to develop more progressive liver pathologies in response to additional metabolic and/or environmental stressors. Mechanistically, this is commonly mediated by the prevalent “two-hit” hypothesis that implies accumulation of TG in hepatocytes (steatosis) in the first hit, followed by triggering progression to inflammation, oxidative stress, and apoptosis in the second hit (22, 35, 36). In more advanced cases, fibrosis is also exacerbated, leading to the progressive form of NAFLD, known as NASH. Environmental stressors [such as high-fat diet (HFD), cigarette smoke, drugs, and pollutants] or metabolic stressors (such as obesity, diabetes, hypertension, hypertriglyceridemia and hypercholesterolemia) are known to trigger progression to the second phase. Nonetheless, the molecular underpinning of steatosis is not well understood. Oxidative stress coupled with hepatocyte apoptosis is believed to play a pivotal role in pathogenesis of NAFLD (22, 37, 38). In fact, emerging data suggest that hepatocyte apoptosis plays a key component in the progression of simple steatosis to NASH (22, 37). Notably, a proof-of-principle, randomized, double blind, placebo-controlled study of GS-9450 (selective inhibitor of caspases 1, 8, and 9) suggests that reducing hepatocellular apoptosis may be a valuable therapeutic strategy in patients with NASH (39).

### Smoking Exacerbates Effects of Dietary Fat on Liver

**Figure 1:**
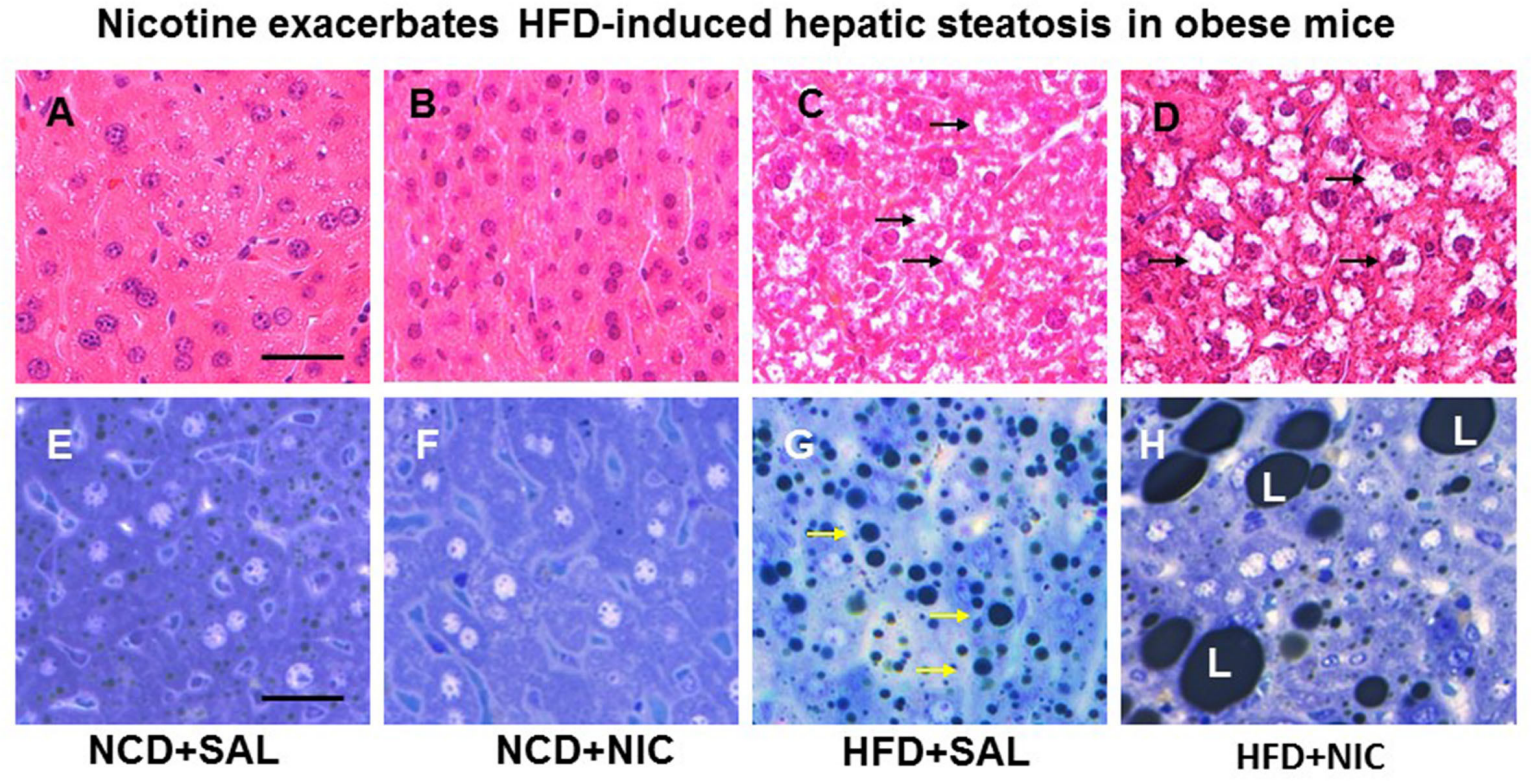
**Representative H&E-stained liver sections from mice fed with normal chow diet (NCD) without (A) or with (B) nicotine exhibit normal histological appearance**. Compared with a mouse on a high-fat diet (HFD), where a modest increase in lipid accumulation (arrow) is detected **(C)**, combined treatment with nicotine and HFD causes a marked increase in lipid accumulation in the liver **(D)**. **(E–H)** Representative light microscopic images of glutaraldehyde-fixed, osmium tetroxide post-fixed, epoxy-embedded, and toluidine-blue-stained live sections from different treatment groups show nicotine plus a HFD **(H)** causes a striking increase in lipid accumulation of varying sizes in hepatocytes compared to those from mice on a HFD alone [**(G)**, arrow]. Mice fed with NCD with **(F)** or without nicotine **(E)** have normal liver morphology. Scale bar = 25 μm [reproduced with permission from Friedman et al. (42)].

Animal experiments using first-hand (delivered *via* a smoking device designed to puff the smoke into the inhalation chamber housing the animals), second-hand smoke (side-stream whole smoke solution delivered *via* a puffer box), or nicotine and models of genetic or diet-induced obesity (DIO) provide perhaps the strongest evidence linking nicotine to hepatic steatosis and NAFLD. Yuan and colleagues (40) demonstrated that HFD-fed apoB100 transgenic mice on C57Bl6J background exposed to second-hand smoke exhibit lipid accumulation in the liver and this effect was mediated by inactivation of AMPK and activation of its downstream target SREBP-1. In another study, Azzalini and colleagues (41) demonstrated that first-hand smoke exacerbates NAFLD in obese Zucker rats. The effect of first-hand smoke on the severity of hepatic steatosis was associated with increased oxidative stress, hepatocyte apoptosis, expression of key genes involved in hepatic fibrogenesis, and inactivation of Akt but stimulation of extracellular signal regulated kinase (ERK) signaling. We used the model of DIO in C57BL6J mice to study the mechanisms underlying the detrimental effects of nicotine and HFD in the development of fatty liver disease (42). Like humans, these mice, when fed a HFD deriving 60% of calories from fat, developed visceral adiposity, hyperglycemia, insulin and leptin resistance, as well as hepatic steatosis (43, 44). We elected to use a single drug (nicotine) as opposed to first-or second-hand smoke in order to eliminate the confounding effects of other components involved in cigarette smoking. Adult C57BL6 male mice were fed a normal chow diet or HFD and received twice daily injections of nicotine (0.75 mg/kg BW, IP) or saline for 10 weeks. Of note, the daily dosage of 1.5 mg/kg BW in mice results in a serum concentration of nicotine that is similar to the clinically relevant concentrations found in habitual cigarette smokers and nicotine-containing chewing gum users (19). We purposely used shorter (10-week) duration to examine the synergistic effects of these two insults in the initiation of NAFLD, as a longer exposure to HFD alone results in extensive steatosis (45) and systemic inflammation (46). We found that nicotine alone did not lead to hepatic steatosis, but it caused hepatic steatosis only when combined with HFD (Figure 1) (42). A significant (*p* < 0.01) increase in the Vv% of lipid droplets together with a reduction in the Vv% of endoplasmic reticulum (ER) (67.8%) and glycogen (49.2%) was also noted in hepatocytes from mice on HFD plus nicotine, compared to mice on HFD alone. The additive effects of nicotine on the severity of HFD-induced hepatic steatosis was associated with significantly greater oxidative stress, increased hepatic TG levels, higher incidence of hepatocellular apoptosis, inactivation (dephosphorylation) of AMPK, and activation of its downstream target ACC (42).

Indeed, these above studies, using various experimental models, demonstrated that nicotine further worsens HFD-induced hepatic steatosis. Summation of the results further indicate that increased oxidative stress and hepatocellular apoptosis, inactivation of Akt and AMPK, and activation of its downstream targets SREBP-1 and ACC, together with stimulation of ERK are involved in the pathogenesis of nicotine plus HFD-induced hepatic steatosis.

### Contribution of Adipose Tissue Lipolysis to Nicotine and HFD-Induced Hepatic Steatosis

**Figure 2:**
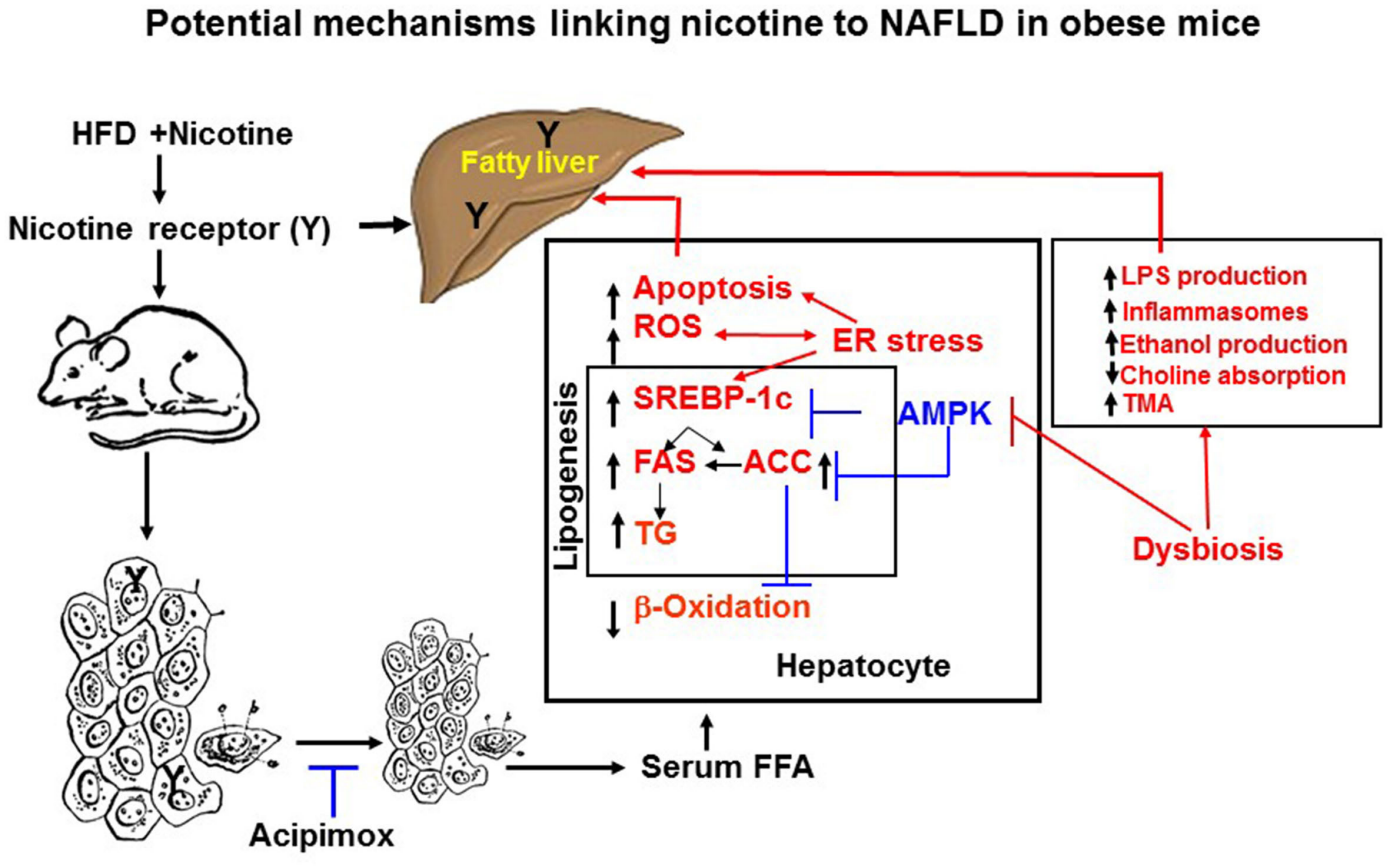
**Potential mechanisms of nicotine plus HFD-induced hepatic steatosis in obese mice**. Nicotine plus a HFD promotes abdominal lipolysis, resulting in free fatty acid (FAA) release from adipose tissue into the circulation, thereby contributing to the buildup of lipids as triglyceride in the liver. In addition, nicotine plus a HFD may also promote *de novo* lipogenesis through inactivation of AMP-activated protein kinase (AMPK) and activation of its downstream target acetyl-coenzyme A-carboxylase (ACC), leading to the development of hepatic steatosis. Inactivation of AMPK can also stimulate lipogenesis through upregulation of key genes in the lipogenic pathway, such as fatty acid synthase (FAS) and ACC, by activating the transcription factor sterol regulatory element binding protein 1 c (SREBP-1c). Intrahepatic lipid accumulation can also trigger hepatocellular apoptosis through generation of oxidative stress coupled with activation of c-Jun NH_2_-termina kinase (JNK)-mediated apoptotic signaling. AMPK inactivation could further sensitize liver cells to nicotine plus HFD-induced apoptosis. There is also growing evidence that chronic endoplasmic reticulum stress through regulation of several pathways leading to oxidative stress, inflammation, perturbed hepatic lipid homeostasis, apoptosis, and autophagy, can also induce hepatic steatosis and its progression to non-alcoholic steatohepatitis. Evidence also suggests a central role of the gut microbiota in obesity and its related disorders, including non-alcoholic fatty liver disease (NAFLD). It is possible nicotine plus a HFD through changes in short-chain fatty acids metabolism, increased intestinal permeability and lipopolysaccharides activation of Toll-like receptors and inflammasomes, endogenous ethanol production, decreased choline availability and increased trimethylamine (TAM) production could cause NAFLD. The multiple mechanisms of nicotine and obesity-induced hepatic steatosis can results from both its nicotinic acetylcholine receptor-mediated and non-receptor effects.

Adipose tissue has the unique function of storing TG in lipid droplets and upon lipolysis, to provide FFA to other organs during time of energy shortage (47). In obesity and other conditions where cellular lipid homeostasis is perturbed, lipolysis can contribute to ectopic lipid accumulation (48). Mounting experimental evidence supports that nicotine considerably decreases HFD-induced adiposity in mice, as determined by dual-energy X-ray absorption densitometry, computed tomography, as well as by magnetic resonance imaging, with no change in lean body mass (19, 49). Nicotine when combined with a HFD, however, significantly increases the levels of serum, hepatic TG, and circulating FFA (19, 42, 50). These results indicate that nicotine in mice on a HFD promotes lipid distribution from adipose tissue to other organs. Decisive evidence that increased adipose tissue lipolysis contributes to nicotine plus HFD-induced hepatic steatosis derives from studies showing that acipimox, an inhibitor of adipose tissue lipolysis, treatment significantly prevented nicotine plus HFD-induced increase in hepatic TG levels and hepatic steatosis (Figure 2) (42). A recent study (19) has also demonstrated that acipimox treatment significantly prevented nicotine plus HFD-induced increase in serum FFA levels and serum and hepatic TG levels, as well as hepatic steatosis (Figure 2). This concept is supported by another evidence showing that inhibition of adipose tissue lipolysis by adipose-specific ablation of desnutrin prevented ectopic lipid accumulation in the liver even when fed with a HFD (51). Together, these results suggest that adipose tissue lipolysis plays a major role in the development of nicotine plus HFD-induced hepatic steatosis.

Mechanistically, nicotine activates AMPKα2 in adipocytes, which phosphorylates MAP kinase phosphatase-1 (MKP1) at serine 334, resulting its proteasome-dependent degradation (19). Nicotine-induced reduction in MKP1, in turn, activates both p38 mitogen-activated protein kinase (p38 MAPK) and c-jun-NH_2_-terminal kinase (JNK), which phosphorylates insulin receptor substrate 1 (IRS1) at serine 307. Phosphorylation of IRS1 leads to its degradation and the subsequent inhibition of Akt, resulting in increased adipose tissue lipolysis and circulating FFA levels (19).

### The Role of ER Stress

Chronic ER stress induces several pathways leading to oxidative stress, inflammation, perturbed hepatic lipid homeostasis, apoptosis, and autophagy that can lead to hepatic steatosis and its progression to NASH [reviewed in Ref. (52)]. ER stress is related with hepatic lipid metabolism by directly increasing lipogenesis and limiting VLDL formation. It has been demonstrated that ER stress contributes to increased hepatic lipogenesis in *ob/ob* mice through SREBP1c activation while overexpression of ER chaperone BIP decreased ER stress and inhibited lipogenesis by inactivating SREBP1 (53). Furthermore, ER stress modulates several factors, including nuclear factor 2 erythroid-related factor 2 (Nrf2), JNK, nuclear factor κB (NF-κB), and c/EBP homologous protein (CHOP), all of which play a role in the inflammatory process, cellular defense against oxidative stress, and cell death. For example, Nrf2 serves as master regular of a cellular defense system against oxidative stress (54, 55). Under physiological conditions, Nrf2 is sequestered in the cytoplasm by Keap1, which facilitates its ubiquitination and proteasomic degradation. Upon exposure to oxidative stress, the sequestration complex brakes down and the dissociated Nrf2 translocates into the nucleus, where it binds to cis-acting antioxidant response elements and promotes the transcription of a large number of cytoprotective genes (56, 57). However, under pathological conditions, such as NASH, NRf2 activity is impaired (52). Consistent with the role of NrF2 in NAFLD, it has been demonstrated that genetic ablation of Nrf2 markedly exacerbates NASH (58). Conversely, enhanced expression of Nrf2 in mice bearing a hepatocyte-specific knockdown of Keap1 attenuated the fatty liver induced by a methionine- and choline-deficient diet (59). JNK is activated in various animal models of obesity and also in patients with NASH and its deletion results in attenuation of fatty liver (22). Activation of JNK has also been documented in HFD-induced hepatic steatosis in apoplipoprotein E knockout mice (60) or nicotine plus HFD-induced hepatic steatosis in obese mice (42). NF-κB is a transcription factor and a primary regulator of inflammatory action. Activation of NF-κB dimers is due to inhibiton of NF-κB kinase (IKK)-mediated phosphorylation-induced proteasomal degradation of IκB, enabling the active NF-κB transcription factor subunits to translocate to the nucleus and induce target gene expression. Persistent activation of NF-κB signaling has been shown in animal models of NAFLD as well as in patients with NASH (35). Furthermore, CHOP plays a pivotal role in ER-induced cell death. Deletion of CHOP decreases hepatocyte apoptosis in alcohol-induced liver disease and reduces cholestsis-induced liver fibrosis (61, 62).

It is worth noting here that both nicotine (63, 64) and HFD (65, 66) are capable of generating hepatic ER stress. Thus, it is possible that nicotine plus HFD could generate severe hepatic ER stress leading to hepatic steatosis. Clearly, further studies are needed to define the role of ER stress in fatty liver disease triggered by nicotine and HFD.

### Connections of Gut Microbiota to NAFLD

Evidence linking dysbiosis (also known as disruption of the normal gut microbiota) contributes to the pathogenesis of NAFLD has accumulated rapidly (67–69). Early studies have shown that patients with biopsy-proven NAFLD had significantly increased gut permeability compared to healthy volunteers (70). Both the increased gut permeability and prevalence of small intestinal bacterial overgrowth correlated with severity of steatosis in the patients with the NAFHD (70). The strongest evidence supporting the role of dysbiosis in NAFLD, however, stems from animals studies where the gut microbiome has been manipulated. It has been shown that microbiome from obese mice is linked to increased energy from the diet and this trail can be transmissible to lean adult germ-free mice by co-housing with obese mice (71). A growing number of studies examining how dysbiosis might drive NAFLD have identified a number of plausible mechanisms, including changes in short-chain fatty acids (SCFAs) metabolism, increased intestinal permeability and lipopolysaccharides (LPS) activation of toll-like receptors (TLRs) and inflammasomes, endogenous ethanol production, decreased choline availability, and trimethylamine production (69). For example, it has been shown that SCFAs can lower FAS activity and hepatic lipid synthesis in HFD-fed mice through activation of AMPK and inactivation of its downstream substrate ACC (72). Evidence exists that smoking can also induce profound changes in intestinal microbiota (73, 74). Taken together, it is possible that nicotine plus a HFD through changes in SCFAs metabolism, increased intestinal permeability and LPS activation of TLRs and inflammasomes, endogenous ethanol production, decreased choline availability and trimethylamine production could cause NAFLD.

## Conclusion and Perspectives

Nicotine when combined with a HFD leads to NAFLD through multiple mechanisms, summarized in Figure 2, including generation of severe oxidative stress and increased hepatocellular apoptosis as well inducing adipose tissue lipolysis resulting in excess delivery of FFA and perturbation of hepatic lipid homeostasis through inactivation of AMPK. There is also growing evidence that chronic ER stress through regulation of several pathways leading to oxidative stress, inflammation, perturbed hepatic lipid homeostasis, apoptosis, and autophagy, can also induce hepatic steatosis and its progression to NASH. Evidence also suggests a central role of the gut microbiota in obesity and its related disorders, including NAFLD. The multiple mechanisms of nicotine and obesity-induced hepatic steatosis is mediated by both its nAChR-mediated and non-receptor effects.

A better understanding of the mechanisms and various diverse signaling pathways responsible for nicotine plus HFD-induced NAFLD may also unveil novel pharmacological targets to treat fatty liver disease and adverse metabolic sequelae. The emerging knowledge about a direct connection of smoking or tobacco products to obesity and fatty liver disease should be considered during the evaluation of regulations on nicotine product manufacturing, distribution, and marketing.

## Author Contributions

AS-H and IS-H conceived and prepared the manuscript. TF critically appraised the manuscript and also wrote a part of the manuscript.

## Conflict of Interest Statement

The authors declare that the research was conducted in the absence of any commercial or financial relationships that could be construed as a potential conflict of interest.
